# Challenges faced by African healthcare workers during the third wave of the pandemic

**DOI:** 10.1002/hsr2.893

**Published:** 2022-10-17

**Authors:** Goodluck Nchasi, Osaretin Christabel Okonji, Rahul Jena, Shahzaib Ahmad, Umar Soomro, Barakat Olajumoke Kolawole, Faisal A. Nawaz, Mohammad Yasir Essar, Abdullahi Tunde Aborode

**Affiliations:** ^1^ Department of Medicine Catholic University of Health and Allied Science Mwanza Tanzania; ^2^ School of Pharmacy University of the Western Cape Cape Town South Africa; ^3^ Bharati Vidyapeeth Deemed University Medical College Pune Maharashtra India; ^4^ King Edward Medical University Lahore Pakistan; ^5^ Department of Medicine Jinnah Medical and Dental College Karachi Pakistan; ^6^ School of Basic Medical Science, Kwara State Polytechnic Kwara State University Malete Nigeria; ^7^ College of Medicine Mohammed Bin Rashid University of Medicine and Health Sciences Dubai United Arab Emirates; ^8^ Department of Dentistry Kabul University of Medical Sciences Kabul Afghanistan; ^9^ Research and Development, Healthy Africans Platform Research and Development Ibadan Nigeria

**Keywords:** Africa, COVID‐19, healthcare workers

## Abstract

Africa experienced the third wave of the coronavirus disease in 2019, which caused an 18% rise in cases in most parts of the continent. As of January 2022, the region had an estimated 10.4 million cumulative cases and more than 233,000 deaths, which add up to the burden on the fragile healthcare system, which continues to face a shortage of staff and resources. In addition, the progression of the pandemic further threatens the supply of healthcare workers in Africa due to the increased risk of infection and death, where more than 10,000 healthcare workers in 40 countries have been infected with the virus. This is amid low vaccination coverage, with only 27% of healthcare workers in Africa being fully vaccinated against the disease. Despite the delayed start and slow progression of the pandemic in Africa, there are increasing concerns over the challenges on the African healthcare workers such as economic insecurity and stressful working conditions, which are associated with limited access to personal protective equipment and other vital resources such as ventilators. In addition, the pandemic further predisposes African healthcare workers to social stigma, burnout, insomnia, depression, and fear of safety in their families. The aim of this study is to highlight the challenges faced by African healthcare workers, provide recommendations for change, and emphasize the need to prioritize their physical and mental well‐being.

## 1 INTRODUCTION

Africa is the second‐largest continent, which covers about one‐fifth of the total land surface area in the world. The emergence of coronavirus Disease 2019 (COVID‐19) caught the world off‐guard, creating havoc in different parts of the globe including Africa. Despite the late start and slow progression of COVID‐19 in Africa, there are increasing concerns over the impacts of the pandemic on the continent.^1^ The emergence of the third wave caused an 18% rise in cases in most parts of the region; this is attributed to the highly transmissible Delta variant, which has been detected in 26 African countries.^2^ In addition, the emergence of the Omicron variant has also escalated the number of COVID‐19 infections, but the number of deaths still remains low in the continent.^3^ Many African countries have developed initiatives and policies such as fostering to channel resources toward preventing transmission of the virus as well as creating presidential task forces to guide the pandemic response.^4^ Despite the increase in COVID‐19 vaccine supplies in Africa through initiatives such as Vaccines Global Access (COVAX), the vaccination rates continue to remain low, with only 10% of the population being fully vaccinated.^4^ As of January 2022, Africa received 500 million vaccine doses and administered about 344 million doses, with the majority of the inoculations in Morocco and Egypt.^5^

The African public health system has historically struggled to provide optimal healthcare services, with facilities being organized in a pyramidal style, in which the best healthcare services are provided by national hospitals, while the village settings offer a weaker health system.^6^ As of January 2022, Africa had an estimated 10.4 million cumulative COVID‐19 cases and more than 233,000 deaths.^7^ This adds up the burden on the continent's fragile healthcare system, which continues to face a shortage of staff and resources. Africa currently has 2.3 healthcare workers (HCWs) per 1000 population. This is significantly low compared to the Americas, which has 24.8 HCWs per 1000 population.^8^ The escalation of the COVID‐19 pandemic combined with inadequate access to personal protective equipment threatens the supply of HCWs in Africa due to the increased risk of infection and deaths, where more than 10,000 health workers in 40 African countries have been infected with the disease amid low vaccine coverage.^9^ Even though most countries in Africa have prioritized health workers as part of their vaccination plans, there continues to be low vaccine coverage, which is attributed to vaccine hesitancy and lack of vaccination services, especially in rural areas and fear of its side effects.^10^, ^11^ This is evident by noting that only 27% of health workers in Africa had been fully vaccinated against COVID‐19, leaving the rest unprotected.^10^ Most of the infections occur among female nurses, who constitute the vast majority of HCWs in Africa.^9^ Sadly, only 27% of health workers in Africa had been fully vaccinated against COVID‐19, leaving the bulk of front lines unprotected.^11^ This is alarming because of the increased risk of COVID‐19 infections to the limited HCWs in Africa, which further deteriorates the health provision capacity in the continent.

The total number of infections in Africa during the third wave of COVID‐19 has greatly impacted the HCWs' mental health due to fear of acquiring the infection, economic insecurity, and stressful working conditions, which are associated with the challenge of limited access to personal protective equipment (PPE) and other equipment such as ventilators. This paralyzing situation is similar to the challenges faced by HCWs in Asia.^11^, ^12^, ^13^, ^14^ Even though both African and Asian HCWs suffer from social stigma, burnout, insomnia, and depression,^11^, ^13^ the primary reason for this challenge in Asia is due to social isolation and a shortage of health personnel to handle the rise in cases of COVID‐19.^13^ Unlike Asia, the perception of pandemic fear in Africa is less intense due to the existence of Ebola outbreaks in West Africa, which has created a relatively prepared community health system that implemented various public health measures to prevent COVID‐19, such as isolating the infected, contact tracing, and early quarantine while patients get tested.^14^, ^15^ The aim of this commentary paper is to expand on the challenges faced by HCWs in Africa during the pandemic, explore the potential implications of not addressing these challenges, and suggest recommendations for alleviating this public health crisis.

## 2 CHALLENGES TO HCWS

The COVID‐19 pandemic has exposed a host of vulnerabilities in the healthcare system of African countries, especially in the wake of the third wave. Disruption of supply chains and inadequate distribution affected access to PPE, isolation beds, and ventilators for HCWs. A pancontinental study found that only 14% of HCWs reported proper access to PPE, 64% reported a lack of isolation wards, and 29% reported no access to ventilators in their work settings.^12^ Reports from South Africa stated that most places had inadequate testing and isolation of patients with COVID‐19, irrespective of disease severity. The hospitals were being stretched to their peak capacities such that critical patients were being treated at home and often had no access to oxygen cylinders, a lifesaving commodity. Most of the hospitals were underprepared to deal with the massive surge of critical patients, contributing to the overall increased mortality, thereby creating a severe toll on the HCW's mental health, resulting in feelings of inadequacy, insomnia, anxiety, helplessness, and physical stress.^16^, ^17^ This burnout as a result of this pandemic has ultimately led to lesser manpower to deal with the pandemic. This is mainly because of widespread fear and anxiety among HCWs leading to hesitancy and dropping out, along with witnessing the loss of fellow HCWs.^16^, ^18^ Social stigmatization and loss of trust in society are other underlooked aspects of the pandemic that take a heavy toll on the HCWs.^17^ Compared to the previous two waves, the third wave has brutally incapacitated the healthcare infrastructure. Most areas in Africa have a fragile healthcare infrastructure as represented by very low availability of intensive care unit (ICU) beds such as that seen in Kenya (600 beds for the population of about 53 million) or poor doctor‐to‐patient ratios (West Africa reported an abysmal two doctors per 10,000 people).^18^ It is no wonder that the third wave has tipped over the healthcare system of Africa.

All of this is worsened by the widespread lack of compliance with COVID‐19 safety protocols due to a lack of political commitment, misinformation and conspiracy theories, and the inaccessibility of government information in remote areas, especially in regions of sub‐Saharan Africa.^18^ Hence, there is a need for good coordination between the public and primary care systems.

One of the key challenges faced by African countries is the emergence of new COVID‐19 variants, which entails the challenge of detecting and controlling the spread of new variants amidst an upsurge in cases.^18^ As seen in several viral endemic diseases such as the seasonal flu, severe acute respiratory syndrome coronavirus 2 virus mutates its genetic sequence, leading to the emergence of novel strains. It is the basis of structural differences in new viral strains that creates differences in transmission rates, infectivity, severity, and associated fatality rate. Downtrodden African regions and countries having low healthcare manpower face a challenge in stepping up measures to detect and control the spread of the evolving variants amidst limited resources.^19^ In July 2021, the Delta variant struck the African healthcare system, thus fueling the hike in the number of Delta variant cases.^18^ This adds further stress to HCWs due to the unknown severity of the new strain and the uncertainty of immune protection offered from prior vaccination. The rapid evolution of the pandemic situation augments the downfall of the preventive healthcare system in Africa due to the inadequacy of surveillance systems.^20^ The threat to African healthcare reaches a whole new level due to the rampant spread of more contagious variants.^20^ The psychological implications of novel mutated strains may aggravate the mental health of the HCWs amidst burnout from extra duty hours and other pandemic management issues.

In Africa, the Omicron variant has now been detected in four countries, with Ghana and Nigeria becoming the first West African countries and the latest on the continent to report the new variant.^21^ So far, Botswana and South Africa have reported 25 and 172 Omicron variant cases, respectively. Globally, more than 20 countries have detected this variant to date. South Africa accounts for 46% of Omicron cases reported globally.^22^ This impacts HCWs in these countries in terms of an ever‐increasing workload. Concomitant outbreaks overburden the pandemic‐stricken healthcare system in Africa. The recent cholera outbreak in the Democratic Republic of Congo has affected 30 people and killed 10 more. This diverts the attention of healthcare from the detection and control of the Omicron variant to curb the cholera outbreak.^23^ Moreover, 70 diseased cases and 35 deaths of yellow fever have been reported in Ghana. The Rift valley fever has recently been declared as an outbreak in Senegal. Chadian health authorities declared a hepatitis E outbreak recently.^24^ Cholera outbreaks in Zimbabwe and Ethiopia, as well as Ebola virus outbreaks in the Democratic Republic of Congo, are alarming threats to the pandemic situation in Africa.^25^ The coinciding outbreaks challenge HCWs in the diagnosis and management of mounting cases of two or more outbreaks.

Vaccine hesitancy has always been one of the prime threats to any successful vaccination program since time immemorial and the COVID‐19 pandemic is no different. A preliminary study conducted in 2021 by the World Health Organization showed that only a measly 27% of the African HCWs were fully vaccinated against COVID‐19.^10^ A cross‐sectional survey of HCWs in Cape Town, South Africa and the Abia State of South‐eastern Nigeria found the prevalence of vaccine hesitancy to be about 41% and 50.5%, respectively, which are shockingly high numbers.^26^, ^27^ Another study among healthcare staff and students from a tertiary care centre in Cape Town, South Africa assessed their vaccine sentiments and found very highly consistent positive vaccine sentiments among both groups, about 90%, and agreement with the vaccine confidence statements were all beyond 90%.^28^ A higher acceptance rate of 90.1% was also found in the Eastern Cape province of South Africa with some variations in the degree of education and professional category.^29^ Cross‐sectional analysis of HCWs from the Democratic Republic of Congo, Ethiopia, and Ghana also found very variable acceptance rates of about 28%, 45.9%, and 70%, highlighting a lot of variation in vaccine acceptance rates.^4^, ^30^, ^31^ What is more interesting than the variability of the acceptance rates among the various regions of Africa are the variations within the same region.^32^ Studies found variable acceptance rates of 21%, 42%, and 24.3% within Egypt itself and fear of side effects, increased risk to HCWs, and lack of clinical trials were cited as the most important factors for the same.^18^, ^33^, ^34^ All the aforementioned studies also assessed socioeconomic factors responsible for hesitancy and some of the most important ones were: age, marital status, profession and income, and occupation.

Addressing hesitancy among HCWs is extremely important since a vaccinated HCWs is much more likely to recommend others to get vaccinated and significantly influences the vaccine uptake of the general population. In the wake of the detection of the newer variants of the virus and its impending threats, the fact that this hesitancy and ignorance is not just limited to the general public but also among HCWs is very concerning and could have disastrous consequences in the long run, and must be addressed on an urgent basis.

In addition, the psychological implications of COVID‐9 are highly pronounced as several studies report adverse impacts on the patients and their families. The families of affected patients confront numerous barriers that negatively impact the overall physical and mental well‐being. Family caregivers of such patients should be provided with information and financial assistance for mitigating common struggles encountered during pandemic care. The concomitant “infodemic” also fuels the existing stress amongst caregivers and calls for improved measures in accessing reliable COVID‐19 knowledge‐sharing resources.^35^ Besides the physical isolation due to quarantine measures, caregivers are often ignored as part of the communication loop, thereby causing a hidden psychological caregiver isolation. Failure to address these challenges can vastly affect the patient care journey by increasing the risk of caregiver burnout coupled with other mental illnesses due to overwhelming distress levels. Hence, there is a need for virtual psychological support sessions among families that are psychologically affected while attending to COVID‐19 patients. This will effectively reduce stress related to psychosocial burden.^36^

## 3 RECOMMENDATIONS

Overall, much more thought is needed about the appropriate utilization of healthcare staff in the COVID‐19 response in rural portions of the continent. During the outbreak, mobile testing units travelled between communities, discovering illness clusters, quarantining affected regions, and erecting makeshift treatment centres as needed. Such efforts aided in directing people and other resources to where they were most needed, yet enabling economic growth in unaffected areas to continue. To identify affected populations that are then targeted for COVID‐19 services, a combination of antigen, antibody, and symptom screening may be required. In the epidemic outbreak such as Ebola, Lassa fever, and so forth, in Africa, testing and then vaccinating HCWs was a major strategy, but testing limits and the lack of a vaccine make that approach challenging in the current pandemic.

Caring for COVID‐19 patients produces a lot of mental stress, which leads to a lot of anxiety and posttraumatic stress disorder, especially among nurses. These circumstances not only have a significant influence on healthcare personnel but also impair their decision‐making abilities and the quality of their interactions with patients. It's important to remember that the stress at work is exacerbated by the same interruptions and uncertainty that the public is experiencing at this moment. It is recommended to provide “relieving programs” for the HCWs that can decimate their mental stress.

Such holistic programs can emphasize on strengthening the HCWs' communication system, sharing accurate and providing timely updates on patient care. This will in turn help prioritize time for the HCWs and incorporate well‐being practices in the workplace. A number of self‐care interventions such as stress reduction techniques, including meditation, mindfulness‐based interventions, and psychologist services, can help build resilience and tackle mental stress and burnout among HCWs. Revision of existing workplace policies is also warranted for improving HCWs' psychological needs across different health systems. It is also critical to recognize that healthcare professionals are potent change agents who should be included in decision‐making and influencing outbreak responses. Healthcare professionals in Africa have a powerful voice in their communities and society at large. They can support local governments and the international community in implementing effective control and treatment programs, readiness initiatives for managing future outbreaks, and public health advocacy for vaccine awareness in the community. Allowing key leadership roles to be led by HCWs may serve as a morale boost in promoting a long‐term HCWs recruitment strategy.

It is recommended to move personnel from other disciplines to medical wards, fast‐tracking medical students into the profession, cancelling HCWs left, and relying on retired health professionals are all examples of initiatives to expand the number of HCWs available to fulfil the burden of care in Africa.

## 4 CONCLUSION

During the COVID‐19 pandemic, African HCWs encountered significant hurdles on social, economic, and health system levels. Therefore, psychology‐based services are crucial to rescue the mental health of HCWs since they are at risk of insomnia and anxiety. However, further research is needed to explore the impact of the increased burden of the pandemic and associated viral variants on the mental health of HCWs, caregivers, and ultimately patient care. The existing challenges require a call for action in strengthening the resources available for the well‐being and safety of HCWs through health policy reforms and governmental support. Failure to address this impending crisis could vastly affect the state of healthcare and future pandemic preparedness in the African region.

## AUTHOR CONTRIBUTIONS

**Goodluck Nchasi**: Writing – original draft; writing – review and editing. **Osaretin Christabel Okonji**: Writing – review and editing. **Rahul Jena**: Writing – original draft; writing – review and editing. **Shahzaib Ahmad**: Writing – original draft; writing – review and editing. **Umar Soomro**: Writing – original draft; writing – review and editing. **Barakat Olajumoke Kolawole**: Writing – original draft; writing – review and editing. **Faisal A. Nawaz**: Conceptualization; writing – review and editing. **Mohammad Yasir Essar**: Conceptualization; writing – review and editing. **Abdullahi Tunde Aborode**: Writing – review and editing.

## CONFLICT OF INTEREST

The authors declare no conflict of interest.

## TRANSPARENCY STATEMENT

The lead author Goodluck Nchasi affirms that this manuscript is an honest, accurate, and transparent account of the study being reported; that no important aspects of the study have been omitted; and that any discrepancies from the study as planned (and, if relevant, registered) have been explained.

## Data Availability

Data sharing is not applicable to this article as no new data were created or analyzed in this study.
